# New automatic algorithm for segmentation of myocardial scar in both inversion recovery and phase sensitive inversion recovery late gadolinium enhancement: validation against TTC and in multi-center, multi-vendor patient data

**DOI:** 10.1186/1532-429X-18-S1-P221

**Published:** 2016-01-27

**Authors:** Jane Tufvesson, Robert Jablonowski, Henrik Engblom, Marcus Carlsson, Anthony H Aletras, Pavel Hoffmann, Alexis Jacquier, Frank Kober, Bernhard Metzler, David Erlinge, Dan Atar, Håkan Arheden, Einar Heiberg

**Affiliations:** 1 Department of Clinical Physiology, Skåne University Hospital in Lund, Lund University, Lund, Sweden; 2 Department of Biomedical Engineering, Faculty of Engineering, Lund University, Lund, Sweden; 3 Laboratory of Medical Informatics, School of Medicine, Aristotle University of Thessaloniki, Thessaloniki, Greece; 4 Deptartment of Cardiology B, Oslo, University Hospital Ullevål and Faculty of Medicine, University of Oslo, University of Oslo, Oslo, Norway; 5 Assistance Publique Hôpitaux de Marseille, Hôpital La Timone, Marseille, France; 6 UMR 7339 CRMBM, Aix-Marseille University, Marseille, France; 7 Department of Cardiology, Medical University of Innsbruck, Innsbruck, Austria; 8 Department of Cardiology, Lund University, Lund, Sweden

## Background

Late gadolinium enhancement (LGE) using magnitude inversion recovery (IR) or phase sensitive inversion recovery (PSIR) has become clinical standard for assessment of myocardial scar. However, there is no clinical standard for quantification of myocardial scar even though multiple methods have been proposed [1]. Simple thresholds have yielded varying results and advanced algorithms have only been validated in single center studies. The weighted algorithm implemented by Heiberg et al. [2] has recently been used by a core lab in two multi-center studies and accounts for both partial volume effects and microvascular obstruction. However, the weighted algorithm was validated in a single center study and PSIR images were not accounted for. Therefore, the aim of this study was to develop an automatic algorithm that accounts for both IR and PSIR LGE images and validate the new algorithm against TTC and multi-center, multi-vendor patient data.

## Methods

The new automatic algorithm was implemented using an intensity threshold defined by expectation maximization (EM) followed by the weighted approach to take partial volume effects into account and improved detection of microvascular obstruction.

The new automatic algorithm and reference delineation in IR and PSIR images was validated against TTC in six pigs with myocardial infarction imaged after seven days of reperfusion. The new automatic algorithm was also validated against reference delineation in 127 patients from the multi-center, multi-vendor studies CHILL-MI and MITOCARE in IR (n = 75) and PSIR (n = 52) images. All patients underwent CMR imaging within 2-6 days following first time ST-elevation myocardial infarction (STEMI) treated with percutaneous coronary intervention (PCI). Reference delineation was performed by a core lab using the original weighted algorithm followed by manual corrections and consensus reading. Analysis was performed using bias (mean ± standard deviation) and linear regression analysis (correlation coefficient). Results are expressed as percent left ventricular mass %LVM.

## Results

**Figure 1:**
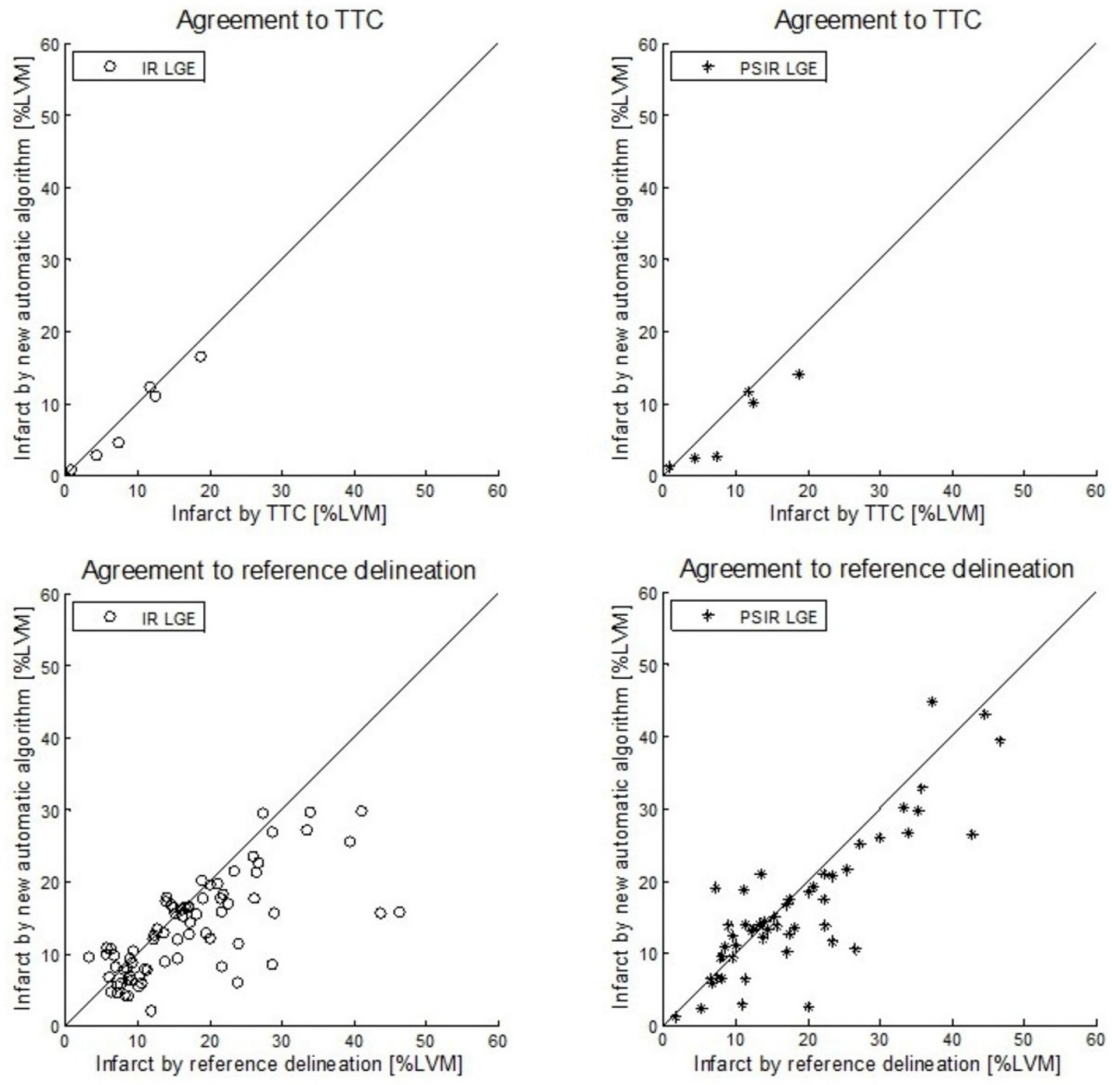
**Validation against TTC and in multi-center patient studies**. Scatter plots of infarct size expressed as % of LVM for the new automatic algorithm against infarct size by TTC in pigs with chronic MI (top row) and against reference delineation in multi-center, multi-vendor patient studies (bottom row) in IR images (o) and PSIR images (*). Solid lines indicate line of identity.

Infarct size by TTC was 9 ± 6 %LVM. Bias to TTC for the new automatic algorithm was -1 ± 1 %LVM and -2 ± 2 %LVM in IR and PSIR images, respectively (Table 1, Figure 1). Infarct size by reference delineation was 17 ± 10 %LVM in patients with IR images and 18 ± 11 %LVM in patients with PSIR images. Bias to reference delineation by the new automatic algorithm was -4 ± 7 %LVM (R=0.72) in IR images and -2 ± 6 %LVM (R=0.86) in PSIR images (Figure 1).

**Table 1:** Validation against TTC in six pigs with chronic myocardial infarction

	IR images		PSIR images	
	Bias to TTC [%LVM]	R-value	Bias to TTC [%LVM]	R-value
New automatic algorithm	-1 ± 1	0.98	-2 ± 2	0.94
Reference delineation	-1 ± 1	0.996	0 ± 0	0.999

Infarct size bias to TTC as % of LVM and correlation R-value for the new automatic algorithm and reference delineation in IR and PSIR images.

## Conclusions

The new automatic algorithm was validated against TTC and in multi-center, multi-vendor patient data with a low bias for both IR and PSIR images. Results show that the new algorithm performs equally well in images acquired with both sequences. The new automatic algorithm can be used as an improved tool for segmentation of myocardial scar in IR and PSIR images.
